# Reliability and Validity of the Multidimensional Scale of Perceived Social Support Among Women and Adolescent Girls With Disabilities in Selected Sub-districts of Bangladesh

**DOI:** 10.7759/cureus.49605

**Published:** 2023-11-28

**Authors:** Munzur E Murshid, Sanmei Chen, Md Moshiur Rahman, Md Ziaul Islam, Yoko Shimpuku, Namira Rahman Era, Santosh Kumar, Mainul Haque

**Affiliations:** 1 Department of Health Sciences, Graduate School of Biomedical and Health Sciences, Hiroshima University, Hiroshima, JPN; 2 Department of Community Medicine, National Institute of Preventive and Social Medicine, Dhaka, BGD; 3 Maternal and Child Health, Independent Practice, Dhaka, BGD; 4 Department of Periodontology and Implantology, Karnavati School of Dentistry, Karnavati University, Gandhinagar, IND; 5 Karnavati Scientific Research Center (KSRC), School of Dentistry, Karnavati University, Gandhinagar, IND; 6 Pharmacology and Therapeutics, National Defence University of Malaysia, Kuala Lumpur, MYS

**Keywords:** the multidimensional scale of perceived social support, bangladesh, girl-children, teenage, females, infirmity, bangla, validity, reliability, mspss

## Abstract

Background

Adequate community-based or societal collaboration and cooperation are considerably important for the overall welfare of women and adolescent girls with disabilities. “The Multidimensional Scale of Perceived Social Support (MSPSS)” has not been evaluated for reliability and validity amid women and adolescent girls with disabilities in the Bangladeshi context.

Methods

A Bangla-translated form of the MSPSS was constructed, and the survey was conducted among 152 women and adolescent girls with disabilities who were purposefully recruited from Bogura Sadar and Chapainawabganj Sadar sub-districts of Bangladesh.

Results

The Cronbach's alpha of the entire scale was 0.868, indicating high internal consistency. Cronbach’s alpha for the family sub-scale was 0.763, the friends sub-scale was 0.820, and the significant others scale was 0.776. The composite reliability for the family sub-scale was 0.849677, the friends sub-scale was 0.881248, and the significant others sub-scale was 0.859668. Convergence reliability was established following sub-scale-wise scores. It affirms the consistency of measurements. The content validity score was >0.62, following the Lawshe approach. The three-factor model was adopted during confirmatory factor analysis when the three-factor model run in SPSS Amos (version 21) CFI (comparative fit index) was 0.919.

Conclusions

In Bangladesh, to the best of our knowledge, our study is initially to calculate the perceived societal assistance of women and adolescent girls with disabilities. We validated the Bangla-translated form of the MSPSS from the Bangladeshi perspective. Researchers and clinicians may rely on our accurate and validated MSPSS translation into Bangla when working with this group. Based on our findings, this study endorses implementing the MSPSS for assessing professed community-based collaboration using the three-factor model, especially among women and adolescent girls with disabilities.

## Introduction

Within the complex and ever-evolving societal framework of Bangladesh, the well-being and social integration of marginalized populations, specifically women and adolescent girls grappling with physical disabilities, represent a critical concern. The influence of social support in shaping their resilience and overall quality of life cannot be overstated, as it provides them with a fundamental cornerstone for navigating the intricate challenges associated with disability and societal expectations. The Multidimensional Scale of Perceived Social Support (MSPSS) [1-4], developed by Gregory Zimet and his team, is a widely used instrument designed to assess an individual's perception of the availability of social support. This scale measures support from three sub-distinct sources: family, friends, and a significant other. Participants respond to items reflecting perceived emotional, informational, and instrumental support from each category. The MSPSS provides a comprehensive understanding of an individual's social support network, offering a nuanced assessment of the various dimensions of support received. Its simplicity and ability to capture diverse sources of support make it a valuable tool in research and clinical settings for evaluating perceived social support across multiple dimensions [1-4]. Nevertheless, the inquiry into the reliability and validity of the MSPSS when explicitly applied to women and adolescent girls with disabilities in selected sub-districts of Bangladesh demands meticulous exploration.

This paper will hopefully serve as a gateway to a comprehensive investigation into the dependability (constancy) and reasonableness (effectiveness) of the MSPSS within the distinctive sociocultural landscape of Bangladesh. Recognizing the central responsibility of community-based collaboration and cooperation in enhancing the breaths of womenfolk and adolescent girls with disabilities, this research endeavors to bridge the existing gaps in our realization of the intricate dynamics of societal accomplishments within this demographic.

Our study aspires to unearth the subtleties of social support within this unique context, recognizing the MSPSS as a potential catalyst for improving their overall well-being and fostering their integration into society. Against the backdrop of selected sub-districts in Bangladesh, this research can potentially significantly subsidize the enduring dialogues on disability rights and social equity in the region, ultimately advancing the cause of empowerment and inclusion for women and adolescent girls with disabilities.

Subsequent chapters in this research will thoroughly examine the research methodology employed. Furthermore, we will explore the implications of our findings for policymakers, practitioners, and the wider academic community. This research may have the potential to influence policy decisions, inform social interventions, and guide further research initiatives, all of which play a pivotal role in shaping the future landscape for women and adolescent girls with disabilities in Bangladesh and, potentially, in similar contexts worldwide.

## Materials and methods

Total participants

One hundred fifty-two women and adolescent girls with disabilities (physical disability 93, partial verbal 24, partial vision 24, partial hearing 11, and aged 11 to 43 years) were included. Physical disability means the person has at least a damaged limb; partial verbal disability means the person can speak but not fluently or clearly like a person without disabilities; partial vision disability means the person’s vision is partially impaired; and partial hearing disability means the person’s hearing capability has not entirely ceased. Their responses in respective domains in the Washington Group short-set questionnaire were utilized to conduct statistical analysis.

Study places

The study was conducted in two sub-districts of Bangladesh, namely Bogura Sadar and Chapainawabganj Sadar Upazila. The sub-districts have been selected purposefully. Both sub-districts are sub-urban, disaster-prone, agriculture-based societies and relatively disadvantaged sub-districts. Additionally, purposive sampling was adopted because the principal investigator (PI) had friends in these sub-districts. These individuals agreed to support PI in collecting data with pleasure. PI is currently a Ph.D. scholar at Hiroshima University without a scholarship.

Study type

The cross-sectional research was performed in April 2023. The “Multi-dimensional Scale of Perceived Social Support” examined recognizes community-based assistance among women and adolescents with disabilities. The reliability and validity process of our utilized tool, the MSPSS English version developed by Zimet et al., Zimet et al., Canty-Mitchell and Zimet, and Dahlem et al. [1-4], was followed (Table 1). There are a few more similar scales, e.g., the Social Support Scale (SSS) [5], the Online Social Support Scale [6], the Family Support Scale (FSS) [7], the Family Resilience Assessment Scale [8], the Family Connectedness Scale [9], the Perceived Support Network Inventory [10], the Sexual Relationship Power Scale [11], and the Social Support Questionnaire - Short Form (SSQ6) [12]. Furthermore, there are different tools to assess social support available at the following link: https://elcentro.sonhs.miami.edu/research/measures-library/social-support-relationship-construct/index.html. Nonetheless, in this study, we are concerned about MSPSS only.

**Table 1:** The Multidimensional Scale of Perceived Social Support Scale Notes: Response level and associated interpretation: Very Strongly Disagree: 1, Strongly Disagree: 2, Mildly Disagree: 3, Neutral: 4, Mildly Agree: 5, Strongly Agree: 6, Very Strongly Agree: 7. “The Multidimensional Scale of Perceived Social Support Scale” was developed by Zimet et al.; Zimet et al.; Canty-Mitchell and Zimet; Dahlem et al. [1-4].

Item	Item description	Response level						
1	There is a special person who is around when I am in need	1	2	3	4	5	6	7
2	There is a special person with whom I can share my joys and sorrows	1	2	3	4	5	6	7
3	My family really tries to help me	1	2	3	4	5	6	7
4	I get the emotional help and support I need from my family	1	2	3	4	5	6	7
5	I have a special person who is a real source of comfort to me	1	2	3	4	5	6	7
6	My friends really try to help me	1	2	3	4	5	6	7
7	I can count on my friends when things go wrong	1	2	3	4	5	6	7
8	I can talk about my problems with my family	1	2	3	4	5	6	7
9	I have friends with whom I can share my joys and sorrows	1	2	3	4	5	6	7
10	There is a special person in my life who cares about my feelings	1	2	3	4	5	6	7
11	My family is willing to help me make decisions	1	2	3	4	5	6	7
12	I can talk about my problems with my friends	1	2	3	4	5	6	7

The translated Bangla MSPSS scale version has been uploaded in appendices and in jpeg format, as shown in Figure 2. The image format has been utilized to facilitate Bengali-speaking authors. The image will retain authentic Bangla font. To the best of our knowledge, this is the first Bangla translation of the MSPSS scale [1-4].

There is no significant difference in the reliability and validity scores of the MSPSS among women and adolescent girls with disabilities in the selected sub-districts of Bangladesh. The “multi-dimensional scale of perceived social support” [1-4] is divided into three sub-scales: “family, friends, and significant others” [1-4].

How to calculate the score

“Significant Other Sub-scale” [1-4]: “Sum across items 1, 2, 5, and 10, then divide by 4."

“Family Sub-scale” [1-4]: “Sum across items 3, 4, 8, and 11, then divide by 4.”

“Friends Sub-scale” [1-4]: “Sum across items 6, 7, 9, and 12, then divide by 4.”

Any mean scale outcome between 1 and 2.9 is perhaps regarded as low community stewardship and protection. At the same time, scores between 3 and 5 could be considered moderate support, and scores between 5.1 and 7 might be regarded as high support [13].

Ethical considerations

The Institutional Review Board of the National Institute of Preventive and Social Medicine (NIPSOM), Dhaka, Bangladesh, has approved the study protocol, Reference No.: NIPSOM/IRB/2023/07, Dated: February 9, 2023. During recruitment, participants provided their informed written consent. Before receiving informed written permission, participants were briefed about the study objectives, procedures, and measures to ensure privacy and anonymity. They are also well-versed in the fact that they could revoke this research regardless of the survey period. The legal guardian provided informed consent on the participant's behalf when she was under 18. All quotes throughout the paper belong to references [1-4].

## Results

The total number of study participants was 152. They were women and adolescent girls with disabilities from selected subdistricts of Bangladesh. There were no missing values in the dataset.

Language validity

The Bangla-translated scale was given to a group of academicians to assess its cultural appropriateness in Bangla and the level of comprehension of each item. The literature mentioned the content validity criteria as 0.62 [14]. Based on these academic experts' opinions and the Lawshe approach, it was concluded that all the scale's items were more than 0.62. No item was removed.

Reliability statistics

Cronbach’s alpha for the full scale was 0.868 (Table 2). Cronbach first evolved alpha in 1951 [15]. It stipulates a portion of the internal consistency of an examination or gradation; it is stated as a number amid 0 and 1 [16-18]. Cronbach's alpha, founded on harmonized and consistent elements, was 0.866. The total number of items was 12 “(item 01, item 02, item 03, item 04, item 05, item 06, item 07, item 08, item 09, item 10, item 11, item 12)” (Table 3) [1-4]. Cronbach’s alpha for the family sub-scale was 0.763. Cronbach's alpha based on standardized items was 0.764. The total number of items was 4: item 03, item 04, item 08, and item 11. Cronbach’s Alpha for Friends Sub-Scale was 0.820. Cronbach's alpha based on standardized items was 0.819. The total number of items was 4: item 06, item 07, item 09, and item 12 (Table 2). Cronbach’s alpha for the significant other sub-scale was 0.776. Cronbach's alpha based on standardized items was 0.782. The total number of items was 4: item 01, item 02, item 05, and item 10 (Table 2).

**Table 2:** The current study - Cronbach’s alpha Notes: Test: reliability analysis; Model: two ways mixed; Type: consistency; confidence interval: 95%. Total item number in full (total scale): 12, sub-scales item number: 4. This analysis was performed based on references [1-4].

In our study - Cronbach’s alpha				
Scale	Family sub-scale	Friend's sub-scale	Significant others sub-scale	Total scale
Score	0.763	0.820	0.776	0.868

**Table 3:** Item statistics Notes: Test: reliability analysis; Model: two ways mixed; Type: consistency; confidence interval: 95%. Total item number in full (total scale): 12, sub-scales item number: 4. The MSPSS English version developed by Zimet and his team during the reliability and validity process was followed [1-4].

Items	Mean (standard deviation)	N
Item 1: “There is a special person who is around when I am in need."	5.64 (0.615)	152
Item 2: “There is a special person with whom I can share joys and sorrows.”	5.50 (0.691)	152
Item 3: “I have friends with whom I can."	5.14 (0.789)	152
Item 4: “I get the emotional help and support I need from my family."	5.13 (0.725)	152
Item 5: “I have a special person who is a real source of comfort to me.”	5.04 (0.868)	152
Item 6: “My friends really try to help me.”	5.11 (0.747)	152
Item 7: “I can count on my friends when things go wrong.”	4.74 (0.638)	152
Item 8: “I can talk about my problems with my family.”	5.01 (0.714)	152
Item 9: “I have friends with whom I can share my joys and sorrows.”	4.94 (0.757)	152
Item 10: “There is a special person in my life who cares about my feelings.”	5.03 (0.849)	152
Item 11: “My family is willing to help me make decisions.”	5.11 (0.756)	152
Item 12: “I can talk about my problems with my friends.”	4.98 (0.818)	152

Among the 12 items, the high response mean was 5.64 in item 01, and the lowest was 4.74 in item 07. Based on the standard deviation score, the most widely spreading response was found in item 05 (SD 0.868), and the least spreading response was in item 01 (SD 0.615). The total number of responses was 152 (Table 2). The mean score of the 12-item scale was 61.36, the variance was 33.065, and the standard deviation was 5.750 (Table 4).

**Table 4:** Scale statistics Notes: Test: Reliability analysis; Model: Two ways mixed; Type: Consistency; Confidence interval: 95%. Total item number in full (total scale): 12, Sub-scales item number: 4.

Mean	Variance	Standard deviation	N of items
61.36	33.065	5.750	12

The item-total statistics table found that overall, Cronbach’s alpha level decreased if any item was deleted (Table 5).

**Table 5:** Item-total statistics Notes: Test: reliability analysis; Model: two ways mixed; type: consistency; confidence interval: 95%. Total item number in full (total scale): 12, sub-scales item number: 4. The MSPSS English version developed by Zimet and team was followed during the reliability and validity statistical analysis process [1-4].

	Scale mean if item deleted	Scale variance if item deleted	Corrected item-total correlation	Squared multiple correlation	Cronbach's alpha if item deleted
Item 1: “There is a special person who is around when I am in need.”	55.72	29.621	0.458	0.520	0.863
Item 2: “There is a special person with whom I can share joys and sorrows.”	55.86	28.628	0.536	0.569	0.858
Item 3: “I have friends with whom I can.”	56.22	27.032	0.659	0.559	0.850
Item 4: “I get the emotional help and support I need from my family.”	56.22	28.215	0.562	0.374	0.857
Item 5: “I have a special person who is a real source of comfort to me.”	56.32	26.535	0.646	0.542	0.851
Item 6: “My friends really try to help me.”	56.25	28.877	0.452	0.511	0.864
Item 7: “I can count on my friends when things go wrong.”	56.62	29.403	0.470	0.430	0.862
Item 8: “I can talk about my problems with my family.”	56.35	28.626	0.514	0.396	0.860
Item 9: “I have friends with whom I can share my joys and sorrows.”	56.41	28.509	0.493	0.582	0.861
Item 10: “There is a special person in my life who cares about my feelings.”	56.32	26.736	0.639	0.602	0.851
Item 11: “My family is willing to help me make decisions.”	56.25	27.871	0.579	0.437	0.856
Item 12: “I can talk about my problems with my friends.”	56.38	27.362	0.589	0.589	0.855

In the correlation matrix, it was found that items are mostly significantly correlated with each other. The significance level of the total score was 0.000 (two-tailed). The Pearson correlation of the total score was 0.540. Critical values for Pearson's correlation coefficient: 0.3104 (significance level 0.01, df=66). The observed value of the total score is greater than the critical value. It denotes the significant validity of the items (Table 6).

**Table 6:** Testing validity of the scale using Pearson correlation coefficient **Correlation is significant at the 0.01 level (two-tailed). *Correlation is significant at the 0.05 level (two-tailed).

		Item 01	Item 02	Item 03	Item 04	Item 05	Item 06	Item 07	Item 08	Item 09	Item 10	Item 11	Item 12	Total score
Item 01	Pearson correlation	1	0.678^**^	0.404^**^	0.345^**^	.0337^**^	0.040	0.228^**^	0.322^**^	0.124	0.315^*^	0.296^*^	0.210^**^	0.540^**^
	Sig. (2-tailed)		0.000	0.000	0.000	0.000	0.623	0.005	0.000	0.127	0.000	0.000	0.010	0.000
	N	152	152	152	152	152	152	152	152	152	152	152	152	152
Item 02	Pearson correlation	0.678^**^	1	0.431^**^	0.357^**^	0.453^**^	0.167^*^	0.195^*^	0.235^**^	0.171^*^	0.390^**^	0.393^**^	0.346^**^	0.619^**^
	Sig. (2-tailed)	0.000	-	0.000	0.000	0.000	0.040	0.016	0.004	0.035	0.000	0.000	0.000	.0000
	N	152	152	152	152	152	152	152	152	152	152	152	152	152
Item 03	Pearson correlation	0.404^**^	0.431^**^	1	0.512^**^	0.524^**^	0.233^**^	0.441^**^	0.539^**^	0.291^**^	0.547^**^	0.408^**^	0.271^**^	0.733^**^
	Sig. (2-tailed)	0.000	0.000	-	0.000	0.000	0.004	0.000	0.000	0.000	0.000	0.000	0.001	0.000
	N	152	152	152	152	152	152	152	152	152	152	152	152	152
Item 04	Pearson correlation	0.345^**^	0.357^**^	0.512^**^	1	0.444^**^	0.243^**^	0.219^**^	0.446^**^	0.268^**^	0.413^**^	0.397^**^	0.295^**^	0.645^**^
	Sig. (2-tailed)	0.000	0.000	0.000	-	0.000	0.003	0.007	0.000	0.001	0.000	0.000	0.000	0.000
	N	152	152	152	152	152	152	152	152	152	152	152	152	152
Item 05	Pearson correlation	0.337^**^	0.453^**^	0.524^**^	0.444^**^	1	0.208^*^	0.306^**^	0.374^**^	0.266^**^	0.663^**^	0.508^**^	0.374^**^	0.730^**^
	Sig. (2-tailed)	0.000	0.000	0.000	0.000	-	0.010	0.000	0.000	0.001	0.000	0.000	0.000	0.000
	N	152	152	152	152	152	152	152	152	152	152	152	152	152
Item 06	Pearson correlation	0.040	0.167^*^	0.233^**^	0.243^**^	0.208^*^	1	0.503^**^	0.185^*^	0.620^**^	0.224^**^	0.226^**^	0.567^**^	0.552^**^
	Sig. (2-tailed)	0.623	0.040	0.004	0.003	0.010	-	0.000	0.023	.0000	0.005	0.005	0.000	0.000
	N	152	152	152	152	152	152	152	152	152	152	152	152	152
Item 07	Pearson correlation	0.228^**^	0.195^*^	0.441^**^	0.219^**^	0.306^**^	0.503^**^	1	0.265^**^	0.461^**^	0.224^**^	0.126	0.371^**^	0.555^**^
	Sig. (2-tailed)	0.005	0.016	0.000	0.007	0.000	0.000	-	0.001	0.000	0.006	0.121	0.000	0.000
	N	152	152	152	152	152	152	152	152	152	152	152	152	152
Item 08	Pearson correlation	0.322^**^	0.235^**^	0.539^**^	0.446^**^	0.374^**^	0.185^*^	0.265^**^	1	0.197^*^	0.480^**^	0.379^**^	0.204^*^	0.603^**^
	Sig. (2-tailed)	0.000	0.004	0.000	0.000	0.000	0.023	0.001	-	0.015	0.000	0.000	0.012	0.000
	N	152	152	152	152	152	152	152	152	152	152	152	152	152
Item 09	Pearson correlation	0.124	0.171^*^	0.291^**^	0.268^**^	0.266^**^	0.620^**^	0.461^**^	0.197^*^	1	0.168^*^	0.266^**^	0.662^**^	0.589^**^
	Sig. (2-tailed)	0.127	0.035	0.000	0.001	0.001	0.000	0.000	0.015	-	0.039	0.001	0.000	0.000
	N	152	152	152	152	152	152	152	152	152	152	152	152	152
Item 10	Pearson correlation	0.315^**^	0.390^**^	0.547^**^	0.413^**^	0.663^**^	0.224^**^	0.224^**^	0.480^**^	0.168^*^	1	0.572^**^	0.392^**^	0.722^**^
	Sig. (2-tailed)	0.000	0.000	0.000	0.000	0.000	0.005	0.006	0.000	0.039	-	0.000	0.000	0.000
	N	152	152	152	152	152	152	152	152	152	152	152	152	152
Item 11	Pearson correlation	0.296^**^	0.393^**^	0.408^**^	0.397^**^	0.508^**^	0.226^**^	0.126	0.379^**^	0.266^**^	0.572^**^	1	0.421^**^	0.663^**^
	Sig. (2-tailed)	0.000	0.000	0.000	0.000	0.000	0.005	0.121	0.000	0.001	0.000	-	0.000	0.000
	N	152	152	152	152	152	152	152	152	152	152	152	152	152
Item 12	Pearson correlation	0.210^**^	0.346^**^	0.271^**^	0.295^**^	0.374^**^	0.567^**^	0.371^**^	0.204^*^	0.662^**^	0.392^**^	0.421^**^	1	0.678^**^
	Sig. (2-tailed)	0.010	0.000	0.001	0.000	0.000	0.000	0.000	0.012	0.000	0.000	0.000	-	0.000
	N	152	152	152	152	152	152	152	152	152	152	152	152	152
Total score	Pearson correlation	0.540^**^	0.619^**^	0.733^**^	0.645^**^	0.730^**^	0.552^**^	0.555^**^	0.603^**^	0.589^**^	0.722^**^	0.663^**^	0.678^**^	1
	Sig. (2-tailed)	0.000	0.000	0.000	0.000	0.000	0.000	0.000	0.000	0.000	0.000	0.000	0.000	-
	N	152	152	152	152	152	152	152	152	152	152	152	152	152

Factor analysis with scale items

Principal component analysis was used as an extraction method. In this factor analysis with 12 items, it was found that only one item can explain the 40.916% variance. That is not satisfactory. When we adopted a higher-order three-factor model, it was found to best fit in the case of factor analysis (Table 7).

**Table 7:** Factor analysis with scale items - total variance elucidated Notes: Total number of items: 12; Extraction method: principal component analysis; Rotation: varimax; Eigenvalues: >1; Absolute value: below 0.10.

Component	Initial eigenvalues			Extraction sums of squared loadings			Rotation sums of squared loadings		
	Total	% of variance	Cumulative %	Total	% of variance	Cumulative %	Total	% of variance	Cumulative %
1	4.910	40.916	40.916	4.910	40.916	40.916	3.339	27.825	27.825
2	1.817	15.142	56.058	1.817	15.142	56.058	2.660	22.168	49.993
3	1.046	8.718	64.777	1.046	8.718	64.777	1.774	14.784	64.777
4	0.956	7.971	72.748	-	-	-	-	-	-
5	0.684	5.698	78.446	-	-	-	-	-	-
6	0.560	4.665	83.111	-	-	-	-	-	-
7	0.426	3.551	86.662	-	-	-	-	-	-
8	0.425	3.541	90.204	-	-	-	-	-	-
9	0.369	3.074	93.278	-	-	-	-	-	-
10	0.326	2.720	95.998	-	-	-	-	-	-
11	0.278	2.316	98.314	-	-	-	-	-	-
12	0.202	1.686	100.000	-	-	-	-	-	-

The Kaiser-Meyer-Olkin (KMO) measure of sampling acceptability or sufficiency [19] for the family sub-scale was 0.770, the friends sub-scale was 0.776, the significant others sub-scale was 0.650, and the total scale was 0.821 (Table 8). The KMO gauge of sampling ampleness is a statistic that shows the proportion of variance in research variables that might be caused by rudimentary features. High values (around 1.0) denote that factor analysis has the benefit of interpreting this research data. If the value is below 0.50, the results of the factor analysis may not be very beneficial [19,20]. Bartlett's test of sphericity was utilized. Maurice Stevenson Bartlett, an English statistician, first developed this test [21,22]. Bartlett’s test of sphericity is cast off to analyze the null proposition that the correspondence matrix is a uniqueness matrix. An identity association matrix interprets surveyed variable stars as orthogonal, discrete, and imperfect for factor analysis [23]. Additionally, Bartlett's sphericity tests revealed that this dataset is seemingly for factor analyses [24]. Thus, Bartlett's test of sphericity assesses whether the correlation matrix of a set of variables helps determine if there are significant relationships among the variables, validating the appropriateness of conducting factor analysis on the dataset. The chi-square for the family sub-scale was 144.543, the friends sub-scale was 219.797, the significant others sub-scale was 215.760, and the total scale was 800.261 (Table 8).

**Table 8:** KMO and Bartlett's test Notes: total number of items in full (total) scale: 12; Total number of items in sub-scale: 4; extraction method: principal component analysis; rotation: varimax; Eigenvalues: >1; absolute value: below 0.10; p=0.000.

	“Family sub-scale”	“Friends sub-scale”	“Significant others sub-scale”	Total 12 items scale
(Kaiser-Meyer-Olkin) measure of sampling adequacy	0.770	0.776	0.650	0.821
(Bartlett's test of sphericity) approx. chi-square	144.543	219.797	215.760	800.261
Degrees of freedom	6	6	6	66
Significance level	0.000	0.000	0.000	0.000
Number of items	4	4	4	12

Factor analysis with sub-scales

When factor analysis runs with a family sub-scale, it was found that component 01 (item 03) has an eigenvalue of more than one and can explain 58.659% of the variance (Table 9).

**Table 9:** Factor analysis with family sub-scale - total variance expounded Notes: Total number of item: 12; Extraction method: principal component analysis, Rotation: varimax; Eigenvalues: >1; Absolute value: below 0.10.

Component	Initial eigenvalues			Extraction sums of squared loadings		
	Total	% of variance	Cumulative %	Total	% of variance	Cumulative %
1	2.346	58.659	58.659	2.346	58.659	58.659
2	0.655	16.380	75.039			
3	0.553	13.832	88.871			
4	0.445	11.129	100.000			

In the factor analysis, when running with the “friends’ sub-scale,” it was found that component 01 (item 06) has an Eigenvalue of more than one and can explain 65.105% variance (Table 10).

**Table 10:** Factor analysis with friends sub-scale - total variance clarified Notes: Total number of item: 12; Extraction method: principal component analysis, Rotation: varimax; Eigenvalues: >1; Absolute value: below 0.10; Extraction method: principal component analysis.

Component	Initial eigenvalues			Extraction sums of squared loadings		
	Total	% of variance	Cumulative %	Total	% of variance	Cumulative %
1	2.604	65.105	65.105	2.604	65.105	65.105
2	0.666	16.639	81.744			
3	0.405	10.115	91.859			
4	0.326	8.141	100.000			

In the factor analysis, when running with significant other sub-scales, it was found that component 01 (item 01) has an Eigenvalue of more than one and can explain 60.520% variance (Table 11).

**Table 11:** Factor analysis with significant others sub-scale - total variance illuminated Notes: Total number of items: 12; Extraction method: principal component analysis, Rotation: varimax; Eigenvalues: >1; Absolute value: below 0.10.

Component	Initial eigenvalues			Extraction sums of squared loadings		
	Total	% of variance	Cumulative %	Total	% of variance	Cumulative %
1	2.421	60.520	60.520	2.421	60.520	60.520
2	0.932	23.308	83.828			
3	0.349	8.721	92.549			
4	0.298	7.451	100.000			

Convergent validity

A convergent validity test was run after adopting a three-factor model. The composite reliability found for the “family sub-scale” [1-4] was 0.849677 (Table 12), the “friends sub-scale” [1-4] was 0.881248 (Table 13), and the significant others sub-scale was 0.859668 (Table 14). A convergent validity test was run after adopting a higher-order three-factor model.

**Table 12:** Composite reliability value for “family sub-scale” Notes: Total item number: 4; Factor loading (FL) achieved from principal component analysis of family sub-scale where rotation was varimax, Eigenvalues: > 1; Absolute value: below 0.10; ME: measurement error, AVE: average variance extracted. This study analysis was conducted based on these references [1-4]. Quote denote references [1-4].

	FL	FL2	ME = (1-F2)	(Total FL)	Total item	AVE	CR
Item 03	0.815	0.664225	0.335775	-	-	-	-
Item 04	0.772	0.595984	0.404016	-	-	-	-
Item 08	0.777	0.603729	0.396271	-	-	-	-
Item 11	0.694	0.481636	0.518364	-	-	-	-
Total	3.058	2.345574	1.654426	9.351364	4	0.586394	0.849677

**Table 13:** Composite reliability value for “friends sub-scale” Notes: Total item number: 4; Factor loading (FL) achieved from principal component analysis of family sub-scale where rotation was varimax, Eigenvalues: > 1; Absolute value: below 0.10; ME: measurement error, AVE: average variance extracted. This study analysis was conducted based on these references [1-4]. Quote denotes references [1-4].

	FL	FL2	ME = (1-FL2)	(Total FL)	Total item	AVE	CR
Item 06	0.841	0.707281	0.292719	-	-	-	-
Item 07	0.700	0.490	0.510	-	-	-	-
Item 09	0.862	0.743044	0.256956	-	-	-	-
Item 12	0.815	0.664225	0.335775	-	-	-	-
Total	3.218	2.60455	1.39545	10.355524	4	0.651138	0.881248

**Table 14:** Composite reliability value for “Significant Others Sub-scale” Notes: Total item number: 4; Factor loading (FL) achieved from principal component analysis of family sub-scale where rotation was varimax, Eigenvalues: >1; Absolute value: below 0.10; ME: measurement error, AVE: average variance extracted. This study analysis was based on references [1-4]. Quotes denote references [1-4].

	FL	FL2	ME = (1-FL2)	(Total FL)	Total Item	AVE	CR
Item 01	0.745	0.555025	0.444975	-	-	-	-
Item 02	0.816	0.665856	0.334144	-	-	-	-
Item 05	0.791	0.625681	0.374319	-	-	-	-
Item 10	0.758	0.574564	0.425436	-	-	-	-
Total	3.110	2.421126	1.578874	9.6721	4	0.605282	0.859668

**Figure 1:**
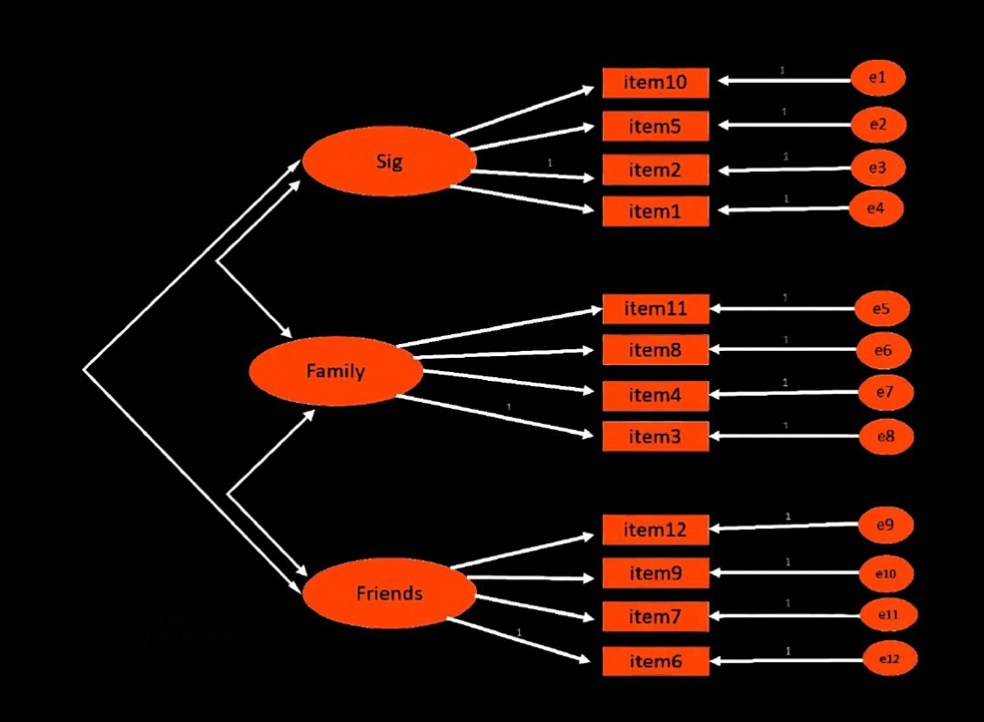
Three-factor model analysis of the current study Noes: Sig: significant others sub-scale, Family: “family sub-scale,” Friends: “friends sub-scale” [1-4]. Testing of the three-factor model utilizing IBM SPSS Amos.

**Figure 2:**
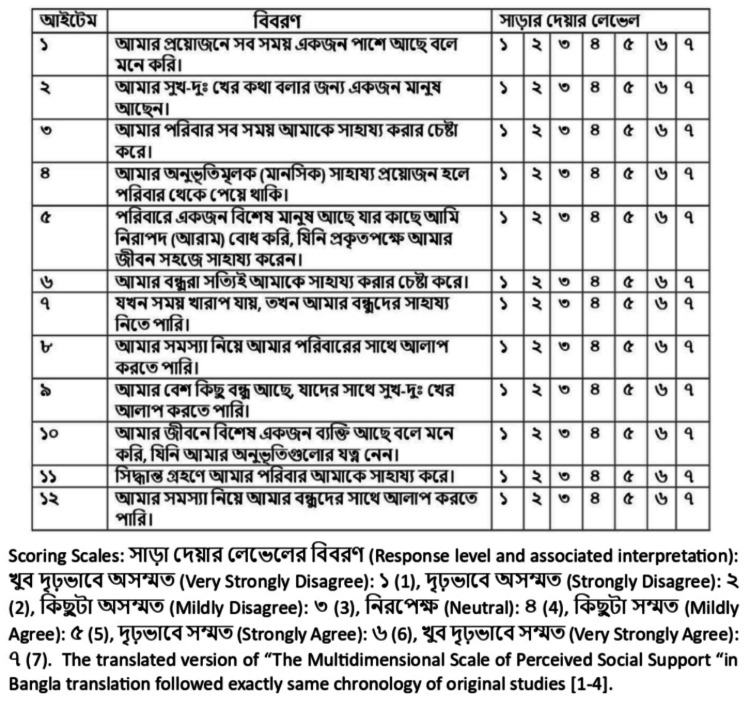
The multidimensional scale of perceived social support Bangla version by the principal investigator and his team We follow the exact scale scores as in the original paper.

In the current study, a three-factor model was adopted. When the factor model was run using IBM SPSS Amos version 21, (IBM Corp. Released 2012, IBM SPSS Statistics for Windows, Version 21.0 (Armonk, NY: IBM Corp.), CMIN (chi-square value) was 111.576, DF (degree of freedom) was 50, CMIN/DF was 2.232, CFI (comparative fit index) was 0.919, and SRMR (standardized root mean squared residual) was 0.068. Figure 1 illustrates a three-factor model analysis.

## Discussion

Descriptive statistics

In our study, the highest response mean among the 12 items was 5.64 in item 01, and the nethermost was 4.74 in item 07. Based on the standard deviation score, the most widely spreading response was found in item 05 (SD 0.868), and the least spreading response was in item 01 (SD 0.615). The total number of responses was 152.

The study conducted by Ebrahim and Alothman [25] in Saudi Arabia revealed that among the 12 items, the high response mean was 3.89 in item 03 (“My family really tries to help me”) [1-4]. The lowest mean was 3.14 in item 12 (“I can talk about my problems with my friends”) [1-4]. Based on the standard deviation score, the most widely spreading response was found in item 12 (SD 1.37), and the least spreading response was in item 03 (SD 1.1). The total number of responses was 606 [25].

The study conducted by Zimet and his team (1998, 1990, 2000, and 1991) found that among the 12 items, the high response mean was 6.22 in item 03, and the lowest mean was 5.38 in item 08 [1-4]. Based on the standard deviation score, the most widely spreading response was found in item 08 (SD 1.51), and the least spreading response was in item 09 (SD 1.01). The total number of responses was 275 [1-4].

The three-factor model and Cronbach’s alpha

Our study explored whether the three-factor model best fits the data set. Cronbach’s alpha for the “family sub-scale" is 0.763 [1-4]; the “friends sub-scale” is 0.820; and significant others are 0.776. Cronbach’s alpha for the total scale was 0.868 [1-4].

The study by Pérez-Villalobos et al. [26] found that the three-factor model best fit their research. They found Cronbach’s alpha for the “family sub-scale” was 0.858 [1-4], for the friends’ sub-scale” 0.941 [1-4], and for the "significant other’s support sub-scale" [1-4], it was 0.873. They did the study among 399 older adults using quote sampling [26].

One more study conducted by Poudel et al. [27] found Cronbach’s alpha for the “family sub-scale” to be 0.75, for the “friends' sub-scale” 0.80, and for the "significant other’s support sub-scale" to be 0.77 [1-4]. The Cronbach’s alpha for the total scale was 0.82. They did the study among 348 adolescents in grades 9 and 10 [27].

Wongpakaran et al. [28] tested MSPSS in Thailand. Among the student group full scale, the Cronbach’s alpha was 0.91, and among recruited patients with major depressive disorders, it was 0.87. They tested the scale among 310 medical pupils from Chiang Mai University and 152 individuals with psychological, emotive, and social ailments [28].

Sharif et al. [29] tested MSPSS in Pakistan. The scale was tested among 1154 pregnant women (older than or equal to 18 years). The MSPSS scale showed excellent internal consistency. The total scale's Cronbach’s alpha level was 0.933. Confirmatory factor analysis retained the three-factor model [19].

One more study conducted among breast cancer survivors by Kim et al. in Korea reported that the Korean-translated MSPSS scale had a Cronbach's alpha score of 0.90. This research also stated the internal consistency was 0.91 for the complete instrument, and the three realms considering partner, household, and acquaintances were 0.96, 0.90, and 0.90, respectively [30].

Convergent validity

The research performed by Wang et al. [21] found that the three-factor model showed the best fit in their analysis. They found that composite reliability for the “family sub-scale” was 0.87, for the “friends’ sub-scale” was 0.88, and for the “significant other’s support sub-scale” was 0.89 [1-4]. This survey was conducted among 487 Chinese paternities of offspring with cerebral palsy [31].

Islam [32] revealed that Cronbach’s alpha for the total scale was 0.860, for the “family sub-scale” was 0.856, the “friends’ sub-scale” was 0.837, and the “significant others sub-scale” was 0.859 [1-4]. Composite reliability for the “family sub-scale” was 0.773, the “friends’ sub-scale was 0.748, and the “substantial others sub-scale” [1-4] was 0.765 [32].

One multicenter study was conducted in Spain among cancer patients (995) from 13 diverse hospitals. The Spanish-language MSPSS instrument had outstanding projected reliability, with scores surpassing 0.90 [23]. Subsequently, the researchers concluded that the Spanish-transformed form of the MSPSS was an acceptable and dependable scale to appraise perceived community assistance among cancer fighters [33].

Another Chinese study among methadone consumers reported that the Cronbach's alpha of the complete MSPSS scale was 0.92 (sub-scales extend: 0.84-0.89) and the intra-class correlation coefficient (ICC, or reliability index [34], is a numeral [35], frequently hinge between 0 and 1) of the total MSPSS questionnaire was 0.65 (sub-scales stretch: 0.57-0.64) [36].

Our study ran a convergent validity test after adopting a higher-order three-factor model. The composite reliability found for the “family sub-scale” was 0.849677, the “friends sub-scale” was 0.881248, and the “significant others sub-scale” was 0.859668 [1-4].

Limitations of this study

One limitation is that the study focuses only on women and girls with disabilities in selected sub-districts of Bangladesh, limiting the ability to generalize the findings to the broader population of women and adolescent girls with disabilities. There is a chance of social desirability bias in the study. To reduce the limitations in the future based on available funding, more study sites with larger sample sizes will be recruited. A pleasant environment will be ensured to minimize social desirability bias, so female counterparts can respond freely without feeling coercion.

## Conclusions

This study provides valuable insights into measuring social support among this demographic. The findings offer a preliminary understanding of the perceived social support levels, which can be a stepping stone for further research and the development of targeted interventions. To strengthen the validity and reliability of the scale in this context, future research should aim for more extensive and diverse samples, consider cultural nuances, and employ longitudinal approaches to track changes over time. Overall, this study lays the foundation for a more comprehensive understanding of social support among women and adolescent girls with disabilities in Bangladesh.
